# The C-terminus of NMDAR GluN1-1a Subunit Translocates to Nucleus and Regulates Synaptic Function

**DOI:** 10.3389/fncel.2018.00334

**Published:** 2018-10-02

**Authors:** Liang Zhou, Jingjing Duan

**Affiliations:** ^1^Department of Pharmacology, College of Pharmaceutical Sciences, Soochow University, Jiangsu, China; ^2^Department of Anatomy and Neurobiology, Zhongshan School of Medicine, SunYat-sen University, Guangzhou, China

**Keywords:** NMDA receptor, GluN1, synapse-to-nucleus communication, synapse transmission, cLTP

## Abstract

NMDARs, the Ca^2+^ permeable channels, play central roles in synaptic plasticity, brain development, learning, and memory. NMDAR binding partners and associated signaling has been extensively studied in synapse-to-nucleus communications. However, whether NMDARs could directly regulate synapse-to-nucleus communications is largely unknown. Here, we analyze the four alternative splicing of the C-terminus isoforms of GluN1 (1a, 2a, 3a, and 4a), and find that C1 domain of GluN1 is necessary for nuclear localization. Besides, we find that the 10 basic amino acids in C1 domain determine the nuclear localization of GluN1 C-terminus. Further investigating the expression patterns of the full length of GluN1 four isoforms shows that only GluN-1a exhibits the cytoplasmic and nucleus distribution in primary hippocampal neurons. Electrophysiological analyses also show that over-expression of GluN1 C-terminus without C1 domain doesn't affect synaptic transmission, whereas GluN1 C-terminus containing C1 domain potentiates NMDAR-mediated synaptic transmission. Our data suggested that the 10 basic amino acids in C1 domain determine translocation of GluN1 C-terminus into nucleus and regulate synaptic transmission.

## Introduction

NMDA receptors (NMDARs) are ionotropic glutamate receptors, which are important for neuronal development, synaptic plasticity, learning, and memory (Tsien et al., 1996). The NMDARs are composed of two GluN1 and two GluN2/3 subunits, which are located at excitatory synapses (Paoletti et al., 2013). The intracellular GluN1 C-terminus contains distinct domains, binds to different proteins, and activates different downstream signaling pathways (Horak and Wenthold, 2009; Gu et al., 2016b). Due to the alternative splicing of the C-terminus, there are four GluN1 isoforms, GluN1-1a, 2a, 3a, 4a (Horak and Wenthold, 2009; Ferreira et al., 2011). The GluN1-1a is the dominant isoform (Laurie and Seeburg, 1994), and the expression levels of GluN1 isoforms are regulated by synaptic activity (Mu et al., 2003; Paoletti et al., 2013).

The synaptic activity modulates the immediate early genes (IEGs) expression in the nucleus (Yang et al., 2014), which is called synapse-to-nucleus communication (Deisseroth et al., 1996, 2003). The synapse-to-nucleus communication is essential for learning and memory (Lim et al., 2017). NMDARs antagonist can block synaptic activation induced IEGs expressions, which demonstrates NMDARs are required for synapse-to-nucleus communication (Mokin and Keifer, 2005; Lonergan et al., 2010). It has been reported that NMDAR associated proteins (Panayotis et al., 2015; Herbst and Martin, 2017) and Ca^2+^ signaling (Hagenston and Bading, 2011) can lead to the activation of downstream signaling pathways during synaptic activity and regulate synapse-to-nucleus communication (Fainzilber et al., 2011; Lim et al., 2017). NMDAR associated proteins, such as Calmodulin (Deisseroth et al., 1998), Jacob (Dieterich et al., 2008), CRTC1 (Ch'ng et al., 2012), JAKMIP1 (Berg et al., 2015), have been extensively studied in synapse-nucleus communication. Calmodulin could translocate to neuronal nuclei upon synaptic stimulation (Deisseroth et al., 1998), and the translocation of calmodulin from cytoplasm to nucleus is mediated by γCaMKII (Ma et al., 2014). The Jacob translocates to neuronal nuclei through binding to importin α upon NMDARs activation (Dieterich et al., 2008), which could be regulated by ERK activity (Karpova et al., 2013). NMDARs activation also promotes the translocation of CRTC1 to neuronal nuclei, which could selectively increase the CREB-mediated transcription upon synapse activity (Ch'ng et al., 2012, 2015; Nonaka et al., 2014). However, whether NMDAR could directly regulate synapse-to-nucleus communication is largely unknown. Here, we found that the C-terminus of GluN1-1a translocates to neuronal nuclei, regulates synaptic transmission. Furthermore, the translocation of GluN1 C-terminus to nuclei could be regulated by neuronal activity.

## Materials and methods

### Plasmids

The plasmids of full-length GluN1-1a,-2a,-3a, and -4a were described preciously (Gu et al., 2016b). C2′oligonucleotides (top and bottom strands) are annealed and directly inserted into pEGFP-N3 vector at Nhe I/Sal I sites. The other truncated and full-length plasmids of GluN1 were created by standard PCR methods: PCR products were amplified from pCAGGS-GluN1-1a,−2a,−3a, and−4a using the relative primers, and subsequently inserted into pEGFP-N3 vector at Nhe I/Sal I sites. All GluN1 mutants were generated by overlapping PCR and cloned into pEGFP-N3 vector at Nhe I/Sal I sites. The primers used for the subclones in this study were provided in the Supplementary Material.

### Cell culture and animals

Human embryonic kidney 293A (HEK293A) cells were cultured in DMEM containing 10% FBS (Gibco). For DNA transfection, Effectene Transfection Reagent (QIAGEN) was used in HEK293 cells, DNA-In® Neuro Transfection Reagent (Thermo Fisher Scientific) was used in primary hippocampal neuron. 24–48 h after transfection, the cells were subjected to immunocytochemistry assay. The mice were housed under the standard conditions of temperature and humidity. All animal procedures in this study were approved by Institutional Animal Care and Use Committee of Soochow University.

### Primary hippocampal neurons culture

Hippocampal primary dissociated neuronal culture was performed as previously described (Gu et al., 2016b). Briefly, the mouse hippocampi were dissected from E18 mouse embryos, and then digested using papain (Worthington) and DNase I (Worthington) to get individual neurons. After centrifuged at 800 rpm for 5 min, the pellet was resuspended in Hanks solution mixed with trypsin inhibitor (Sigma) and BSA, and then centrifuged at 800 rpm for 10 min. The pellet was resuspended in Neurobasal plating media containing 2% FBS, 2% B27 supplements, and 2 mM L-glutamine. Hippocampal neurons were plated at 200,000 cells/well on coverslips coated with poly-D-lysine in 24-well plates. Neurobasal culture media containing 2% B27 supplements, and 2 mM L-glutamine were used to replaced the culture media each other day.

### Organotypic hippocampal slice culture

The mouse hippocampi were dissected from P6–P8 wild-type mice as previously described (Gu et al., 2016a), and transfected biolistically with plasmids in DIV3-4. Slices were cultured for an additional 2 days before recording. For recording evoked EPSCs in slice cultures, the extracellular solution is artificial cerebrospinal fluid (ACSF) containing (in mM) KCl 2.5, CaCl_2_ 4, MgCl_2_ 4, NaH_2_PO4 1.25, NaHCO_3_ 25, glucose 7, sucrose 210. 15 μM 2-chloroadenosine and 100 μM picrotoxin were added into ACSF to dampen epileptiform activity and block the GABA_A_ receptors. The supplier of pharmacological reagents is Abcam.

### Neuronal stimulation

DIV 12–14 dissociated hippocampal neurons were used to perform neuronal stimulation experiments. After bath application of the relative chemicals, all samples were transferred to the solution without the related reagents for 15–20 min, and then the samples were subjected to immunocytochemistry assay. For neuronal membrane depolarization induction, 50 mM KCl was used to treat hippocampal neurons for 5 min. For NMDARs activation induction, 100 μM NMDA (Abcam) and 2 μM glycine was given by bath application for 5 min. For chemical long-term potentiation (cLTP) induction, 200 μM glycine in ACSF was briefly applied for 3 min to stimulate synaptic NMDARs, which is described as previously study (Lu et al., 2001). For chemical long-term depression (cLTD) induction, 50 μM NMDA (Abcam) was given by bath application for 3 min. Ten micromoles MG-132 (Sigma) was used to block the proteasome. Fifty micromoles DHPG (Abcam) was given by bath application for 5 min to induce group 1 metabotropic glutamate receptor-mediated LTD.

### Immunocytochemistry assay

HEK293A cells or hippocampal neurons grown on coverslips were rinsed with PBS twice and fixed in 4% paraformaldehyde (PFA) for 15 min at room temperature, permeabilized with 0.2% TritonX-100 in PBS for 5 min, after blocking with 5% normal goat serum (NGS) in PBS for 1 h, the primary antibodies anti-GluN1 (mouse,1:1,000, NeuroMab, N308/48), anti-GluN1 (rabbit, 1:1,000, Millipore, AB9864) were used at room temperature for 3 h, and then the second antibodies Alexa Fluor 405-conjugated goat anti-mouse and Alexa Fluor 555-conjugated goat anti-rabbit (Invitrogen, Molecular Probes) were incubated for 1 h. Coverslips were mounted with Fluoromont G (Southern Biotech). Fluorescent images were acquired on a Zeiss510 laser scanning confocal microscope and Olympus IX71 inverted microscope with identical settings for each group. For image quantification analysis, maximal projection images were generated by LSM510 browser software, the integrated fluorescent intensity of GluN1 was measured with ImageJ software (NIH, Bethesda). Statistical analysis was conducted with GraphPad Prism6 software using one-way analysis of variance (ANOVA).

### Synaptic electrophysiology

The AMPA EPSCs and NMDA EPSCs were recorded by stimulating Schaffer collaterals pathway with monopolar glass electrodes filled with ACSF. GFP-positive pyramidal neurons at the CA1 region were visualized with GFP fluorescence. GFP-positive neuron and the neighboring GFP-negative neuron were chose to perform the paired whole-cell recordings. AMPA EPSCs were recorded at −70, and NMDA EPSCs were recorded at +40 mV. The NMDA EPSCs were measured at 100 ms after stimulation. The components of intracellular solution were (in mM) CsMeSO_4_ 135, NaCl 8, HEPEs 10, Na_3_GFP 0.3, MgATP 4, EGTA 0.3, QX-314 5, and spermine 0.1. The 3–5 MΩ borosilicate glass pipettes were used for recording.

### Statistics

All data were given as mean ± s.e.m. Statistical significance between means was calculated using Student's *t*-test.

## Results

### C1 domain of GluN1 C-terminus is necessary for nuclear translocation

**Figure 1:**
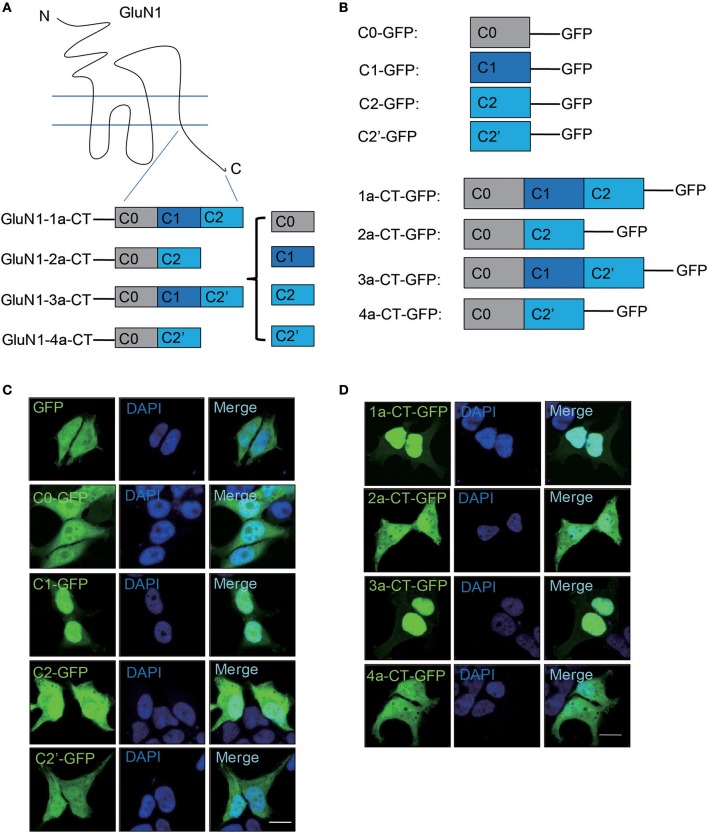
C1 domain determines the nuclear localization of GluN1 C-terminus in HEK293A cells. **(A)** Schematic diagram of GluN1 C-terminus. **(B)** The plasmids of truncated GluN1 C-terminus were used in this study. **(C)** HEK293A cells were transfected with GFP, C0-GFP, C1-GFP, C2-GFP, or C2'-GFP for 24 h, and then the cells were subjected to immunocytochemistry assay. DAPI was used to visualize the cell nuclei. Scale bar: 10 μm. **(D)** The C-terminus of four GluN1 isoforms were transfected in HEK293A cells for 24 h, and then the cells subjected to immunocytochemistry assay. DAPI was used to visualize the cell nuclei. Scale bar: 10 μm.

Our previous study shows the C-terminus of GluN1 play a crucial role in GABAergic synapses formation (Gu et al., 2016b), indicating that the C-terminus of GluN1 is important for NMDARs-mediated biological functions. Due to alternative splicing of the C-terminus, there are four GluN1 isoforms, GluN1-1a, 2a, 3a, and 4a (Figure 1A). They are composed of four domains, C0, C1, C2, and C2' (Figure 1B). Firstly, we investigated the cellular distribution of these four domains in HEK293A. The results showed that C1-GFP mainly localized in nucleus, while the other domains showed the whole cell diffuse distribution (Figure 1C). To verify whether C1 domain is required for nuclear localization, we over expressed 4 isoforms of GluN1 C-terminus in HEK293A cells and primary hippocampal neurons. We found that only the 1a-CT-GFP and 3a-CT-GFP containing the C1 domain showed nuclear localization in HEK293A cells (Figure 1D) and primary hippocampal neurons (Supplementary Figure S1). Thus, our data demonstrated that C-terminus of GluN1 containing C1 domain was necessary for nuclear translocation.

### Ten basic amino acids in C1 domain determines the nuclear localization of GluN1 C-terminus

**Figure 2:**
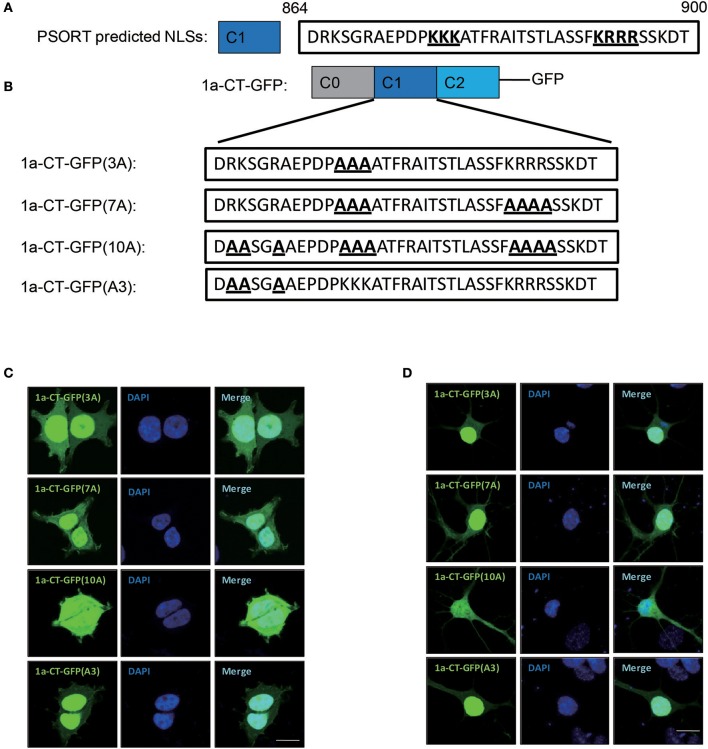
Ten amino acid in C1 domain determines the nuclear localization of GluN1 C-terminus. **(A)** the protein subcellular localization prediction tool (PSORT) predicted nuclear localization signal in C1 domain. **(B)** Indicated mutation for 1a-CT-GFP (3A), 1a-CT-GFP (7A), 1a-CT-GFP (10A), or 1a-CT-GFP (A3). **(C,D)** The HEK293A (**C**) and primary hippocampal neurons **(D)** were transfected with 1a-CT-GFP (3A), 1a-CT-GFP (7A), 1a-CT-GFP (10A), or 1a-CT-GFP (A3) for 24 h, and then the cells were subjected to immunocytochemistry assay. DAPI was used to visualize the cell nuclei. Scale bar: 10 μm.

Previously, it is suggested that C1 domain contains a bi-particle nuclear localization signal (NLS) by the protein subcellular localization prediction tool (PSORT) (Holmes et al., 2002; Jeffrey et al., 2009) (Figure 2A). To verify whether the C1 domain contains NLS, we constructed several mutants of 1a-CT (Figure 2B). We found that the bi-particle 7 basic amino acids [1a-CT-GFP(7A)] were not necessary for the nuclear localization of GluN1 C-terminus, but the 10 basic amino acids in the C1 domain 1a-CT-GFP(10A) regulated the nuclear localization of GluN1 C-terminus in HEK293A cells and primary hippocampal neurons (Figures 2C,D).

### GluN1 C-terminus translocates to nucleus in primary neurons

**Figure 3:**
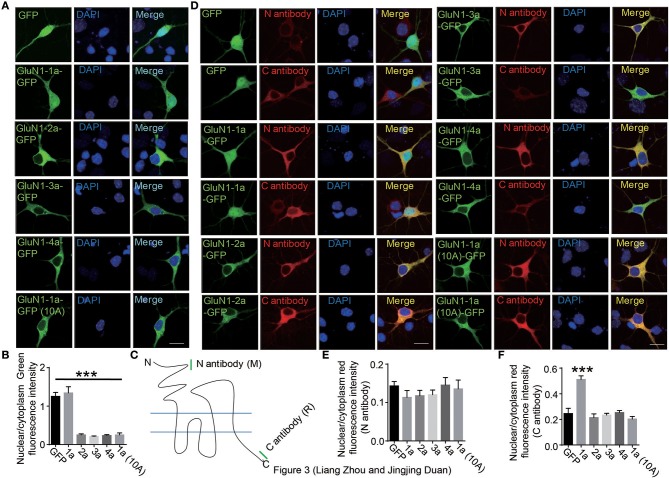
The C-terminus of GluN1-1a translocates to nucleus in primary hippocampal neuron. **(A)** The primary hippocampal neurons were transfected with GFP, GluN1-1a-GFP, GluN1-2a-GFP, GluN1-3a-GFP, GluN1-4a-GFP, or GluN1-1a-GFP (10A) for 24 h, and then the cells were subjected to immunocytochemistry assay. DAPI was used to visualize the cell nuclei. Scale bar: 10 μm. **(B)** Quantitative data (mean ± SEM) of **(A)** from three independent experiments. ***P < 0.001, Student's *t*-test. **(C)** Two GluN1 antibodies were used in this study. The mouse N antibody recognizes the N terminus amino acids 42–361 of GluN1 extracellular domain, and the rabbit C antibody recognizes the C2 domain of GluN1. **(D)** The primary hippocampal neurons were transfected with GFP, GluN1-1a-GFP, GluN1-2a-GFP, GluN1-3a-GFP, GluN1-4a-GFP, or GluN1-1a-GFP (10A) for 24 h, and then stained with the N and C antibodies of GluN1 for immunofluorescence measurement. DAPI was used to visualize the nuclei. Scale bar: 10 μm. **(E,F)** Quantitative data (mean ± SEM) of (**D**) from three independent experiments. ***P < 0.001, Student's *t*-test.

To explore the nuclear localization of full-length GluN1, we transfected the full-length four isoforms of GluN1 fused to GFP into cells. Our data showed that the full-length of GluN1 showed diffuse cytoplasmic distribution in HEK293A cells (Supplementary Figure S2). In primary hippocampal neurons, we found that the full-length of GluN1-1a exhibited cytoplasmic and nuclear distribution, while the GluN1-2a, GluN1-3a, GluN1-4a, and GluN1-1a(10A) isoforms showed diffuse cytoplasmic distribution (Figures 3A,B). Given that single GluN1 subunit could not form the functional NMDARs in HEK293A cells (Cao et al., 2011; Hansen et al., 2014), we transfected GluN1 and GluN2A subunits in HEK293A cells to examine whether GluN2 is required for the translocation of GluN1-1a C terminus, the results showed the full-length GluN1-1a still showed cytoplamic distribution in HEK293A cells (Supplementary Figure S3), which suggests that the intrinsic genetic determinants of neuronal form determine the translocation of GluN1 C-terminus to nucleus in primary hippocampal neurons.

There were three possibilities to explain the whole cell diffuse distribution of GluN1-1a-GFP. Firstly, the full-length of GluN1-1a-GFP could translocate to nucleus because of artificial phenomena of over-expression; secondly, the GFP protein can be detached or auto-cleaved from GluN1-1a-GFP in neurons for some unknown reasons; the third possibility is that the C-terminus of GluN1-1a can be detached or cleaved from GluN1-1a-GFP in neurons, so the C-terminus contained GFP fused C1 domain can translocate to nucleus. To test these hypotheses, two commercial antibodies were used (Figure 3C). The N-terminus antibody is a mouse antibody that can recognize the N-terminus of GluN1 extracellular domain (amino acids 42–361); the C-terminus antibody is a rabbit antibody that can only recognize the C2 domain part of GluN1 C-terminus (LQNQKDTVLPRRAIEREEGQLQLCSRHRES) (Figure 3C). To test the specificity of the antibodies, we over expressed the full-length of GluN1-1a, 2a, 3a, and 4a in HEK293A cells. The results showed that N antibody could recognize all four full-length isoforms, whereas the C antibody could only recognize the full-length GluN1-1a and GluN1-2a containing C2 domain (Supplementary Figure S4A). We also used the C-terminus of four isoforms to test these two commercial antibodies. The results showed the N antibody couldn't recognize the C-terminus, while the C antibody could recognize the GluN1-1a-CT and 2a-CT containing C2 domain (Supplemental Figure S4B). Therefore, our data showed the two antibodies could specifically recognize the immunogenic regions.

In cultured hippocampal neurons, the immunocytochemistry assays showed that the nuclear fluorescence of GluN1-1a-GFP could be identified by the C antibody (Figures 3D–F). Because the C antibody also recognizes the endogenous GluN1, the red fluorescence in the nucleus is weaker than in the cytoplasm (Figure 3D). Thus, our data support the third hypothesis that C-terminus of GluN1-1a can be detached or cleaved from GluN1-1a-GFP in neurons, and the C-terminus including GFP can translocate to nuclei.

### Chemical LTP regulates the translocation of C-terminus of GluN1 to nucleus

**Figure 4:**
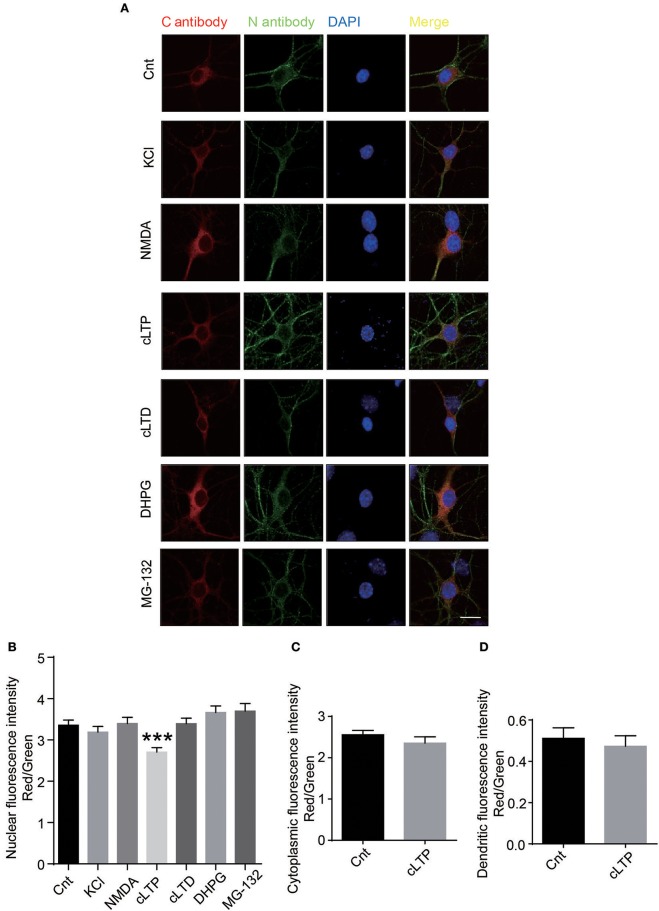
Neuronal activity regulates the cleavage of GluN1. **(A)** The primary hippocampal neurons were treated with several reagents to activate the different signal pathways. 50 mM KCl was used to depolarize the neuronal membrane. One hundred micromoles NMDA (Abcam) and 2 μM glycine was used to activate NMDARs. Two hundred micromoles glycine was used to induce cLTP. Fifty micromoles NMDA was used to induce cLTD. Fifty micromoles DHPG was used to induce group 1 metabotropic glutamate receptor-mediated LTD. Ten micromoles MG-132 was used to block the proteasome. **(B)** The quantitative data were presented with the nuclear fluorescence intensity of GluN1 by measuring ratio of nuclear red fluorescence (rabbit C antibody) to nuclear green fluorescence (mouse N antibody). (Number of neurons in each group: Cnt: *n* = 39, KCl: *n* = 38, NMDA: *n* = 36, cLTP: *n* = 42, cLTD: *n* = 41, DHPG: *n* = 48, MG-132: *n* = 35). Student's t test, ***P < 0.001. **(C,D)** The quantitative data was presented with the cytoplasmic fluorescence intensity of GluN1 **(C)** or dendritic fluorescence intensity **(D)** by measuring ratio of cytoplamic or dendritic red fluorescence (rabbit C antibody) to cytoplamic or dendritic green fluorescence (mouse N antibody). (Number of neurons in each group: Cnt: *n* = 39, cLTP: *n* = 42). Student's *t*-test, cytoplamic fluorescence *p* = 0.2899, dendritic fluorescence *p* = 0.5989.

To explore the relationship of nuclear translocation and neuronal activity, we treated the neurons with KCl to depolarization the neuron, NMDA and glycine to activate NMDARs, glycine to induce chemical LTP (cLTP), NMDA to induce chemical LTD (cLTD), MG-132 to block the proteasome, DHPG to induce group 1 metabotropic glutamate receptor-mediated LTD. Using C antibody to detect C-terminus of GluN1, N antibody to detect total GluN1, we calculated the nuclear fluorescence'ratio of C antibody to N antibody. The results show that the chemical long-term potentiation (cLTP) treatment could significantly decrease the nuclear fluorescence intensity of GluN1 (Figures 4A,B).There is no significant difference in the ratios of cytoplasmic and dendritic fluorescence intensity after cLTP treatment (Figures 4C,D). Taken together, these data demonstrates that cLTP could affect the translocation of GluN1 C-terminus to nucleus.

### The C-terminus of GluN1-1a potentiates glutamate transmission

**Figure 5:**
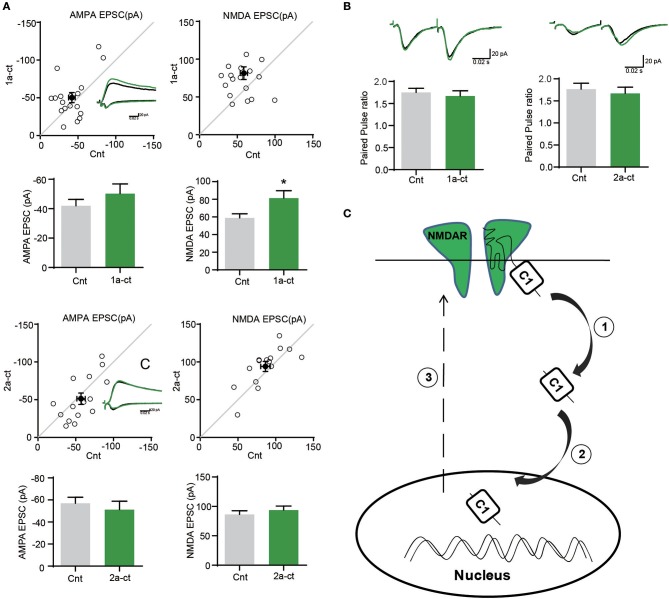
The C terminus of GluN1 regulates synaptic functions. **(A)** The AMPA EPSCs and NMDA EPSCs were recorded after over expressing the 1a-CT-GFP or 2a-CT-GFP. (Amplitude of AMPA EPSCs: Cnt, 41.97 ± 4.403; 1a-CT-GFP, 50.24 ± 6.659, *n* = 18. *p* = 0.1911; Amplitude of NMDA EPSCs: Cnt, 58.84 ± 4.795; 1a-CT-GFP, 81.42 ± 8.345, n = 19. *p = 0.0169 < 0.05; Amplitude of AMPA EPSCs: Cnt, 57.05 ± 5.471; 2a-CT-GFP, 51.26 ± 7.547, *n* = 15. *p* = 0.3183; Amplitude of NMDA EPSCs: Cnt, 86.42 ± 6.226; 2a-CT-GFP, 94.00 ± 6.685, *n* = 15. *p* = 0.1146; Amplitude of AMPA EPSCs: Cnt, 34.38 ± 4.166). **(B)**There were no differences in PPRs after over expressing 1a-CT-GFP or 2a-CT-GFP. (PPR control, 1.750 ± 0.09609 and PPR 1a-CT-GFP, 1.673 ± 0.1146, *n* = 14, *p* = 0.3147; PPR control, 1.767 ± 0.1343 and PPR 2a-CT-GFP, 1.673 ± 0.1380, *n* = 13, *p* = 0.4804). **(C)** A schematic diagram showing the translocation of GluN1 C-terminus to neuronal nucleus. The GluN1-1a C-terminus containing C1 domain could be cleaved by protease ①, and then translocates to neuronal nucleus ②, which eventually potentiates NMDAR-mediated synaptic transmission ③.

Given the crucial role of NMDARs in synaptic functions, we then investigated the regulation of GluN1-1a C terminus on synaptic transmission. We transfected GluN1 C-terminus into neurons, and then did the electrophysiological recording. We found that over-expression of 2a-CT-GFP without C1 domain didn't change AMPA EPSCs or NMDA EPSCs, whereas 1a-CT-GFP containing C1 domain could potentiate NMDAR-mediated synaptic transmission (Figure 5A). No change of paired-pulse ratio (PPR) of GluN1-1a-CT-GFP and GluN1-2a-CT-GFP suggested that C1 domain of GluN1 did not affect presynaptic neurotransmitter release probability (Figure 5B).

## Discussion

NMDARs are widely expressed in neurons throughout the central nervous system with distinct pharmacological and electrophysiological properties because of diversity of subunit composition (Paoletti et al., 2013; Hansen et al., 2014). Once the synaptic plasticity happens, the neuronal nucleus must be informed to activate the IEGs expression, which is mediated by the synapse-to-nucleus signaling pathway (Deisseroth et al., 1996, 2003). Several proteins binding to NMDARs or forming the complex with NMDARs in the postsynaptic density have been reported that can relay the information between synapses and nucleus, such as Calmodulin (Deisseroth et al., 1998), nuclear factor-κB (NF-κB) (Meffert et al., 2003), importins (Ch'ng and Martin, 2011), and Jacob (Dieterich et al., 2008).

Here we found that NMDARs subunit GluN1-1a involved in synapse-to-nucleus communication. Our results showed that the C-terminus of GluN1-1a could translocate to neuronal nucleus (Figures 1–3) and the 10 basic amino acids in C1 domain determined the nuclear localization of GluN1 C terminus. The electrophysiological recording showed that C1 domain of GluN1 affected the NMDAR-mediated synaptic transmission (Figure 5). Taken together, these data indicated that the 10 basic amino acids in C1 domain determined the translocation of GluN1 C-terminus into neuronal nucleus, and this translocation is highly related to synaptic transmission (Figure 5).

In our study, although the GluN1-1a and GluN1-3a both contained C1 domain in the C-terminus, only the GluN1-1a showed nuclear fluorescence in neurons (Figure 3), which suggested that the adjacent alternatively spliced C2'domain affects this process, as the C2' domain can suppresses the function of C1 domain (Standley et al., 2000).

The neuronal activity regulates the localization of NMDARs (Rao and Craig, 1997). Here, we found that cLTP regulated the translocation of C-terminus of GluN1 (Figure 4). However, the proteins that lead to the detachment or cleavage of C-terminus from the full-length of GluN1-1a are needed to be further identified. The calcium-dependent protease calpain could be a candidate, as calpain and NMDARs can regulate the functions of each other (Hell et al., 1996; Wu et al., 2005; Szydlowska and Tymianski, 2010). The LTP coordinates the homeostasis of synapses (Vitureira and Goda, 2013) and normalization of synapses (Hardingham et al., 2007), whether the translocation of GluN1-1a C-terminus to neuronal nuclei mediates the LTP associated these physiological functions, which are needed to be further investigated.

In summary, we found that the C-terminus of GluN1-1a can translocate to neuronal nuclei, and regulate the synaptic activity, which could be affected by cLTP.

## Author contributions

LZ supervised the research. LZ and JD performed the experiments. LZ and JD wrote and revised the manuscript.

### Conflict of interest statement

The authors declare that the research was conducted in the absence of any commercial or financial relationships that could be construed as a potential conflict of interest.
