# Supplementary material for: The C-terminus of NMDAR GluN1-1a Subunit Translocates to Nucleus and Regulates Synaptic Function
Source: Front Cell Neurosci. 2018 Oct 2;12:334. doi: 10.3389/fncel.2018.00334 (PMC6176477; doi:10.3389/fncel.2018.00334)
Supplement: Supplementary file 1 [file Table_1.doc]

**Supplementary Materials**

Primers used for subclones of GluN1 plasmids.

Four truncated domains (C0, C1, C2 and C2′) primers:

C0-GFP:

Forward: 5’- TCGGCTAGCACCATGATCGCCTACAAGCGACACAAGGATGCC -3’,

Reverse: 5’- AGCTGTCGACCTGCAGGTTCTTCCTCCACACGTTCACGGCTG-3’

C1-GFP:

Forward: 5’-TCGGCTAGCACCATGGATAGAAAGAGTGGTAGAGCAGAGCCCGAC -3’,

Reverse: 5’- AGCTGTCGACCGTGTCTTTGGAGGACCTACGTCTCTTG-3’

C2-GFP:

Forward: 5’- TCGGCTAGCACCATGAGCACCGGGGGTGGACGCGGCGCTTTG -3’,

Reverse: 5’- AGCTGTCGACGCTCTCCCTATGACGGGAACACAGCTGCAG-3’

C2′-GFP:

Forward: 5’- TCGGCTAGCACCATGCAGTACCATCCCACTGATATCACGGGCCCGCTCAACCTCTCAGATCCCTCGGTCAGCACCGTGGTGGTCGACAGCT -3’,

Reverse: 5’- AGCTGTCGACCACCACGGTGCTGACCGAGGGATCTGAGAGGTTGAGCGGGCCCGTGATATCAGTGGGATGGTACTGCATGGTGCTAGCCGA-3’

Four cytoplasmic tails of GluN1 primers:

1a-CT-GFP:

Forward: 5’- TCGGCTAGCACCATGATCGCCTACAAGCGACACAAGGATGCC -3’,

Reverse: 5’- AGCTGTCGACGCTCTCCCTATGACGGGAACACAGCTGCAG-3’

2a-CT-GFP:

Forward: 5’- TCGGCTAGCACCATGATCGCCTACAAGCGACACAAGGATGCC -3’,

Reverse: 5’- AGCTGTCGACGCTCTCCCTATGACGGGAACACAGCTGCAG-3’

3a-CT-GFP:

Forward: 5’- TCGGCTAGCACCATGAGCACCGGGGGTGGACGCGGCGCTTTG -3’,

Reverse: 5’- AGCTGTCGACCACCACGGTGCTGACCGAGGGATCTGAG-3’

4a-CT-GFP:

Forward: 5’- TCGGCTAGCACCATGAGCACCGGGGGTGGACGCGGCGCTTTG -3’,

Reverse: 5’- AGCTGTCGACCACCACGGTGCTGACCGAGGGATCTGAG-3’

Four full-length GluN1 primers:

GluN1-1a-GFP:

Forward: 5’- TCGGCTAGCACCATGAGCACCATGCACCTGCTGACA -3’,

Reverse: 5’- AGCTGTCGACGCTCTCCCTATGACGGGAACACAGCTGCAG-3’

GluN1-2a-GFP:

Forward: 5’- TCGGCTAGCACCATGAGCACCATGCACCTGCTGACA -3’,

Reverse: 5’- AGCTGTCGACGCTCTCCCTATGACGGGAACACAGCTGCAG-3’

GluN1-3a-GFP:

Forward: 5’- TCGGCTAGCACCATGAGCACCATGCACCTGCTGACA -3’,

Reverse: 5’- AGCTGTCGACCACCACGGTGCTGACCGAGGGATCTGAG-3’

GluN1-4a-GFP:

Forward: 5’- TCGGCTAGCACCATGAGCACCATGCACCTGCTGACA -3’,

Reverse: 5’- AGCTGTCGACCACCACGGTGCTGACCGAGGGATCTGAG-3’

GluN1 mutants primers:

3A (KKK-AAA):

Forward: 5’- GTGGTAGAGCAGAGCCCGACCCTGCAGCGGCAGCCACATTTAGGGCTATCACCTC -3’,

Reverse: 5’- AGGGTCGGGCTCTGCTCTACCACTCTTTCTATCCTGCAG -3’

7A (KKK-AAA, and KRRR-AAAA):

Forward: 5’-CACCCTGGCCTCCAGCTTCGCGGCAGCTGCGTCCTCCAAAGACACG -3’,

Reverse: 5’- GAAGCTGGAGGCCAGGGTGGAGGTGATAGCCCTAAATGTGGCTGCCGCTGCAGGGTCGGGCTC-3’

A3 (RKSGR-AASGA):

Forward: 5’- GAACGTGTGGAGGAAGAACCTGCAGGATGCAGCGAGTGGTGCAGCAGAGCCCGACCCT -3’,

Reverse: 5’- CTGCAGGTTCTTCCTCCACACGTTCACGGCTGCAAAAGCCAGCTGCATCTG-3’

10A (KKK-AAA, KRRR-AAAA, and RKSGR-AASGA):

Forward: 5’- GAACGTGTGGAGGAAGAACCTGCAGGATGCAGCGAGTGGTGCAGCAGAGCCCGACCCT -3’,

Reverse: 5’- CTGCAGGTTCTTCCTCCACACGTTCACGGCTGCAAAAGCCAGCTGCATCTG-3’

**Supplementary Figures**

|  |
| --- |

**Supplementary Figure S1.** The 1a-CT-GFP and 3a-CT-GFP exhibit the nuclear localization in primary hippocampal neuron. The primary hippocampal neurons were transfected with 1a-CT-GFP, 2a-CT-GFP, 3a-CT-GFP, or 4a-CT-GFP for 24h, and then the cells were subjected to immunocytochemistry assay. DAPI was used to visualize the cell nucleus. Scale bar: 10 μm.

|  |
| --- |

**Supplementary Figure S2.** The full-length of GluN1 shows the diffuse cytoplasmic distribution in HEK293A cells. HEK293A cells were transfected with GluN1-1a-GFP, GluN1-2a-GFP, GluN1-3a-GFP, GluN1-4a-GFP, or GluN1-1a (10A)-GFP for 24h, and then the cells were subjected to immunocytochemistry assay. DAPI was used to visualize the cell nucleus. Scale bar: 10 μm.

**Supplementary Figure S3.** The full-length of GluN1-1a shows the cytoplasmic distribution in HEK293A cells. HEK293A cells were co-transfected with GFP/GluN1-1a-GFP and Myc/Myc-GluN2A for 24h, and then the cells were subjected to immunocytochemistry assay. DAPI was used to visualize the cell nucleus. Scale bar: 10 μm.

**Supplementary Figure S4.** Functional validation of GluN1 antibodies. (A). HEK293A cells were transfected with GFP, GluN1-1a-GFP, GluN1-2a-GFP, GluN1-3a-GFP, or GluN1-4a-GFP for 24h, and then the cells were subjected to immunocytochemistry assay using the N and C antibodies. DAPI was used to visualize the cell nucleus. Scale bar: 10 μm. (B). HEK293A cells were transfected with 1a-CT-GFP, 2a-CT-GFP, 3a-CT-GFP, or 4a-CT-GFPfor 24h, and then the cells were subjected to immunocytochemistry assay using the N and C antibodies. DAPI was used to visualize the cell nucleus. Scale bar: 10 μm.
